# 889. The age of RSV cases changes consistently over the course of annual epidemics: potential insights into RSV transmission dynamics based on surveillance data from seven countries.

**DOI:** 10.1093/ofid/ofad500.934

**Published:** 2023-11-27

**Authors:** Jean-Sebastien Casalegno, Ana Paula Rodrigues, Vernon Lee, Cheryl Cohen, Q Sue Huang, Alfredo Bruno Caicedo, Raquel Guiomar, Li Wei Ang, Tim Cook, Domenica de Mora, Mathieu Bangert, Rolf Kramer, Lisa Staadegaard, Susanne Heemskerk, Jojanneke van Summeren, Adam Meijer, John Paget, Saverio Caini, Anne Teirlinck

**Affiliations:** Virology Department, Institut des Agents Infectieux, Hôpital de la Croix-Rousse, HCL, Lyon, France, Lyon, Rhone-Alpes, France; Instituto Nacional de Saúde Doutor Ricardo Jorge, Lisbon, Portugal., Lisbon, Lisboa, Portugal; Ministry of Health, Singapore., Singapore, Not Applicable, Singapore; Centre for Respiratory Disease and Meningitis, National Institute for Communicable Diseases, Johannesburg, South Africa., Johannesburg, Gauteng, South Africa; Institute of Environmental Science and Research Limited (ESR), National Centre for Biosecurity and Infectious Disease (NCBID), Upper Hutt, New Zealand., Upper Hutt, Wellington, New Zealand; Instituto Nacional de Investigación en Salud Pública (INSPI), Centro de Referencia Nacional de Influenza y otros Virus Respiratorios, Guayaquil, Ecuador., Guayaquil, Guayas, Ecuador; Instituto Nacional de Saúde Doutor Ricardo Jorge, Lisbon, Portugal., Lisbon, Lisboa, Portugal; Ministry of Health, Singapore., Singapore, Not Applicable, Singapore; Institute of Environmental Science and Research Limited (ESR), National Centre for Biosecurity and Infectious Disease (NCBID), Upper Hutt, New Zealand., Upper Hutt, Wellington, New Zealand; Instituto Nacional de Investigación en Salud Pública (INSPI), Centro de Referencia Nacional de Influenza y otros Virus Respiratorios, Guayaquil, Ecuador., Guayaquil, Guayas, Ecuador; Sanofi Vaccines, Lyon, France, Lyon, Rhone-Alpes, France; Sanofi Vaccines, Lyon, France, Lyon, Rhone-Alpes, France; Netherlands Institute for Health Services Research (NIVEL), Utrecht, theNetherlands., Utrecht, Utrecht, Netherlands; Netherlands Institute for Health Services Research (NIVEL), Utrecht, theNetherlands., Utrecht, Utrecht, Netherlands; Netherlands Institute for Health Services Research (NIVEL), Utrecht, theNetherlands., Utrecht, Utrecht, Netherlands; Centre for Infectious Diseases Research, National Institute for Public Health and the Environment (RIVM), Bilthoven, the Netherlands, Bilthoven, Utrecht, Netherlands; Netherlands Institute for Health Services Research (NIVEL), Utrecht, theNetherlands., Utrecht, Utrecht, Netherlands; Netherlands Institute for Health Services Research (NIVEL), Utrecht, theNetherlands., Utrecht, Utrecht, Netherlands; Centre for Infectious Diseases Research, National Institute for Public Health and the Environment (RIVM), Bilthoven, the Netherlands, Bilthoven, Utrecht, Netherlands

## Abstract

**Background:**

RSV infections affects people of all ages but the seasonality of RSV among different age groups have not been thoroughly examined. In this study, we investigated changes in the age distribution of RSV cases during the course of annual epidemics.

**Methods:**

We analyzed surveillance data (2008-2019) from the Netherlands, the city of Lyon (France), Portugal, Singapore, Ecuador, South Africa, and New Zealand using the Global Epidemiology of RSV (GERi) study database. We defined annual RSV seasons as the period from July to June in the Northern hemisphere, or the calendar year in the tropics and Southern hemisphere. Within each season, we divided the data into “epidemic quartiles”, corresponding to each quartile of RSV cases in the season. We used multilevel multinomial logistic regression models with season as the higher-level variable to assess whether the likelihood of RSV cases being aged < 1 year or ≥5 years (vs. those aged 1 to < 5 years) changed over time during the season.

**Results:**

A total of 27,391 RSV cases were included. Across all countries, the odds of RSV cases being aged < 1 year (vs. 1 to < 5 years) were significantly higher in the fourth epidemic quartile compared to the first quartile; the relative risk ratio (RRR) ranged between 1.35 in Lyon, France, and 2.56 in the Netherlands. Similarly, the odds of RSV cases being aged ≥5 years (vs. 1 to < 5 years) were significantly higher in the fourth epidemic quartile compared to the first quartile (except in Singapore); the RRR ranged from 1.75 in Ecuador to 6.70 in Lyon, France. The results were consistent when stratifying the data by level of care (primary vs. secondary) or using a lower cut-off of six months of age.

**Conclusion:**

The age distribution of RSV cases changes over the course of a season, with infants (< 1 year) and older children (≥5 years), adults, and elderly people constituting a higher proportion of RSV cases in the later phases of annual epidemics. These findings provide potential insights into the transmission dynamics of RSV and may inform prevention and control measures, e.g. the implementation of targeted age-specific interventions. **Funding**: This research was funded by a collaborative agreement with Sanofi / AstraZeneca and is part of the RODEO research program for RSV disease.

**Disclosures:**

**Mathieu Bangert, PhD**, Sanofi: Staff member **Rolf Kramer, n/a**, Sanofi: Stocks/Bonds **John Paget, n/a**, Sanofi Pasteur: Grant/Research Support

